# Genome-wide association study of *Gossypium arboreum* resistance to reniform nematode

**DOI:** 10.1186/s12863-018-0662-3

**Published:** 2018-08-03

**Authors:** Ruijuan Li, John E. Erpelding, Salliana R. Stetina

**Affiliations:** 1 Present address: Department of Plant Biology, University of California, Davis, One Shields Avenue, Davis, CA 95616 USA; 2 USDA-ARS, Crop Genetics Research Unit, 141 Experiment Station Road, PO Box 345, Stoneville, MS 38776 USA

**Keywords:** Cotton, Genome-wide association study, Genotyping-by-sequencing, Germplasm, *Gossypium arboreum*, Host-plant resistance, reniform nematode, Single nucleotide polymorphism

## Abstract

**Background:**

Reniform nematode (*Rotylenchulus reniformis*) has emerged as one of the most destructive root pathogens of upland cotton (*Gossypium hirsutum*) in the United States. Management of *R. reniformis* has been hindered by the lack of resistant *G. hirsutum* cultivars; however, resistance has been frequently identified in germplasm accessions from the *G. arboreum* collection. To determine the genetic basis of reniform nematode resistance, a genome-wide association study (GWAS) was performed using 246 *G. arboreum* germplasm accessions that were genotyped with 7220 single nucleotide polymorphic (SNP) sequence markers generated from genotyping-by-sequencing.

**Results:**

Fifteen SNPs representing 12 genomic loci distributed over eight chromosomes showed association with reniform nematode resistance. For 14 SNPs, major alleles were shown to be associated with resistance. From the 15 significantly associated SNPs, 146 genes containing or physically close to these loci were identified as putative reniform nematode resistance candidate genes. These genes are involved in a broad range of biological pathways, including plant innate immunity, transcriptional regulation, and redox reaction that may have a role in the expression of resistance. Eighteen of these genes corresponded to differentially expressed genes identified from *G. hirsutum* in response to reniform nematode infection.

**Conclusions:**

The identification of multiple genomic loci associated with reniform nematode resistance would indicate that the *G. arboreum* collection is a significant resource of novel resistance genes. The significantly associated markers identified from this GWAS can be used for the development of molecular tools for breeding improved reniform nematode resistant upland cotton with resistance introgressed from *G. arboreum*. Additionally, a greater understanding of the molecular mechanisms of reniform nematode resistance can be determined through genetic structure and functional analyses of candidate genes, which will aid in the pyramiding of multiple resistance genes.

**Electronic supplementary material:**

The online version of this article (10.1186/s12863-018-0662-3) contains supplementary material, which is available to authorized users.

## Background

Reniform nematode (RN, *Rotylenchulus reniformis*) is an obligate, semi-endoparasitic nematode that feeds on the root system of upland cotton (*Gossypium hirsutum*) causing significant yield losses [1, 2]. The United States ranks third in worldwide cotton production with 2.8 million metric tons (MMT) produced in 2016 (http://apps.fas.usda.gov/psdonline/psdDataPublications.aspx). Yield losses from RN typically range from 1 to 5% in the southeastern cotton producing states [2] with an estimated loss of 24,211 MT in 2015 valued at approximately US$36 million [3]. According to Robinson [1], RN has replaced root-knot nematode (*Meloidogyne* spp.) as the major pathogenic nematode of cotton in the mid-south region of United States. RN infection in cotton is initiated when vermiform preadult females penetrate the stele of the roots, where successful parasitism results in the establishment of a multi-nucleus syncytium inside the nematode feeding cells, which serves as the sole nutrient source [1, 2]. Symptoms of RN infestation include plant stunting, suppressed root growth, and reduced yield; but these symptoms are typically uniform across the field making it difficult to visually assess nematode damage [2].

As the most widely planted cotton species, *G. hirsutum* is a natural allotetraploid (genome AADD referred to as 2(AD)_1_) that likely resulted from the interspecific hybridization between diploid ancestors of *G. arboreum* (genome A_2_) and *G*. *raimondii* (genome D_5_) [4, 5]. Nevertheless, no RN resistant *G. hirsutum* cultivars have been identified to effectively manage the nematode [1, 6, 7]. Instead, related cotton species are being used as a source of resistance. RN resistance has been identified in at least 10 of the 50 cotton species, including nine diploid species *G. anomalum* (genome B_1_B_1_), *G. herbaceum* (genome A_1_A_1_), *G*. *raimondii* (genome D_5_D_5_), *G*. *somalense* (genome E_2_E_2_), *G*. *stocksii* (genome E_1_E_1_), *G. thurberi* (genome D_1_D_1_) [8], *G. arboreum* (genome A_2_A_2_) [8–10], *G*. *aridum* (genome D_4_D_4_) [11], *G*. *longicalyx* (genome F_1_F_1_) [8, 12], and one allotetraploid species *G. barbadense* (genome AADD referred to as 2(AD)_2_) [7, 8, 13]. Among them, only *G*. *longicalyx* showed immunity to RN [8, 12]. Introgression of resistance from *G*. *longicalyx* into *G. hirsutum* was successful [12] resulting in the release of two breeding lines, LONREN-1 (PI 669509) and LONREN-2 (PI 669510) [14]. However, severe root necrosis and progressive root mass loss under high levels of RN inoculum have been reported for the *G*. *longicalyx* source of resistance, which are typical of hypersensitive responses [15]. This root damage results in severe plant stunting, poor growth rate, and reduced lint yields [16], which has hindered the use of the *G*. *longicalyx* source of resistance in cotton improvement programs. RN resistance from *G*. *aridum* and *G. arboreum* has also been introgressed into *G. hirsutum* [10, 11]; however, the difficulties of introgressing and developing cultivars with stable resistance from these other cotton diploid species have slowed progress in cultivar development. Greater success has been achieved with RN resistance derived from *G. barbadense*; although, few *G. barbadense* germplasm accessions showed resistance [1, 6–8]. Currently, RN resistance from two *G. barbadense* germplasm accessions (PI 163608 and PI 608139) has been introgressed into *G. hirsutum* with the release of breeding lines [17–20]. To increase the diversity of RN resistance, additional resistant cultivars derived from different resources are required and the *G. arboreum* germplasm collection has been shown to be a major source of resistant accessions [8, 21].

Studies of the genetic inheritance of RN resistance introgressed from *G*. *longicalyx* identified one dominantly inherited gene that conferred resistance, which was localized to the A sub-genome of *G. hirsutum* on chromosome 11 (*Ren*^*lon*^) and flanked by codominant simple sequence repeat (SSR) marker BNL3279_114 [22]. The RN resistance derived from *G. barbadense* was governed by three quantitative trait loci (QTL), *Ren*^*barb1*^ and *Ren*^*barb2*^ on chromosome 21 and *Ren*^*barb3*^ on chromosome 18, that showed a D sub-genome origin with each having a significant additive and dominance effect [13]. Recently, Wubben et al. [23] reported that QTL *Ren*^*barb1*^ and *Ren*^*barb2*^ on chromosome 21 can be resolved to a single locus (*Ren*^*barb2*^). They also showed that the majority of RN resistance was conferred by *Ren*^*barb2*^ and that locus *Ren*^*barb3*^ did not confer resistance in the absent of *Ren*^*barb2*^; although, the two QTL were required to show expression of resistance similar to the *G. barbadense* parental accession. Locus *Ren*^*barb2*^ associated with the same SSR marker (BNL3279_109) [13] reported for locus *Ren*^*lon*^ on chromosome 11. These regions on chromosomes 11 and 21 are putative homeologous regions [24] and resistance genes for several other cotton disease were associated with these regions [25–28]. RN resistance derived from *G*. *aridum* was also of D sub-genome origin and mapped to a single locus (*Ren*^*ari*^) on *G. hirsutum* chromosome 21 flanked by two SSR markers BNL3279_132 and BNL2662_090 [11]. QTL *Ren*^*ari*^ and *Ren*^*barb2*^ may be allelic [13]; whereas, *Ren*^*ari*^ and *Ren*^*lon*^ are possibly duplicated genes on homeologous regions of the *G. hirsutum* genome [24]. Thus, marker BNL3279 that showed different amplicon sizes was common among the three different RN resistance sources. DNA sequences of bacterial artificial chromosome clones [28] and RNA-seq [29] quantification of genes close to marker BNL3279 suggested that resistance (*R*) genes, including leucine-rich repeat (LRR) receptor-like kinases and nucleotide binding site-LRR domain-containing protein, could be associated with RN resistance.

The traditional approach for mapping genes responsible for a biological trait is family-based linkage mapping commonly using bi-parental populations, which capture the recombination events between chromosomes inherited from two inbred strains. This approach has been widely used in cotton improvement programs to determine genomic regions harboring genes for disease resistance, fiber yield, and lint quality traits in order to identify markers linked to these traits for marker-assisted breeding [30, 31]. While this method is powerful to map the causative genomic locations for various traits specific to a defined population, the low genomic resolution and time required to generate a population for more precise mapping limits its application [32]. In comparison, a Genome-Wide Association Study (GWAS) exploits all the historical recombination present in a natural population or in a collection of germplasm accessions. By employing the non-random association (LD, linkage disequilibrium) between alleles at unique loci along the chromosomes, GWAS is able to identify the causative marker-trait associations and/or associations in LD with the causative loci, given sufficient genome-wide markers are provided. Thus, with GWAS, a higher genomic resolution can be reached and more alleles, which are not present in the mapping parents used for the family-based method, can be captured, although rare alleles associated with important traits might be lost [33].

GWAS has been conducted in cotton to identify SSR markers associated with fiber quality traits [34] and Verticillium wilt resistance [35]. These studies had limitations for both mapping resolution and genome coverage, because of the availability of a small number of SSR markers for genotyping. Next generation sequencing technology has greatly increased the number of markers available for genotyping. Genotyping-by-sequencing (GBS), which employs reduced representation sequencing strategy, can generate genome-wide single nucleotide polymorphism (SNP) markers at a low cost and high efficiency [36]. Alternately, a CottonSNP63K array with over 45 k putative intraspecific and over 17 k interspecific SNP markers was recently released [37]; although, the costs associated with this technology remain high. Application of these powerful technologies and resources in GWAS will help the advancement of genetic studies of different traits of cotton including RN resistance.

In an effort to utilize new sources of RN resistance identified from the *G. arboreum* germplasm collection [21] and dissect the genetic basis of this resistance, this study genotyped 246 *G. arboreum* accessions for GWAS of RN resistance. The aims were (i) to detect QTL and SNPs associated with *G. arboreum* RN resistance, and (ii) to identify candidate resistance genes in the associated genomic regions. The detected SNPs from this study can serve as important tools in marker-assisted breeding for the introgression of resistance and the development of *G. hirsutum* cultivars with improved RN resistance and to increase the diversity of resistance. Additionally, the candidate genes identified from this study will help in understanding and evaluation of the molecular mechanisms of resistance to improve the utilization of RN resistance sources.

## Methods

### Evaluation of reniform nematode infection response of *G. arboreum* accessions

An association mapping panel consisting of 246 *G. arboreum* accessions (Additional file 1: Table S1) was selected from the USDA National Plant Germplasm System cotton collection (https://npgsweb.ars-grin.gov). These accessions represent landraces and cultivars that were donated to the collection before 2001 [38]. To minimize within-accession variation, self-pollinated bolls from one plant were selected for each accession. These self-pollinated seeds were used to evaluate nematode resistance and for DNA isolation. The reniform nematode infection response for the accessions was evaluated in growth chamber tests maintained at 28 °C using a 16 h photoperiod as described by Stetina and Erpelding [21]. Briefly, accessions were planted in plastic pots (Ray Leach SC10U Cone-tainer, Stuewe and Sons Inc., Tangent, OR) containing a steam pasteurized soil mixture composed of two parts sand and one part sandy loam soil. One seedling representing an individual accession was maintained in each pot and pots were arranged in a completely randomized experimental design with three replications. The *G. hirsutum* accession PI 529251 (cv. Deltapine 16) was included as a susceptible control [8]. The *G. arboreum* accession PI 615699 was used as a resistant control [9] and was included in the mapping panel. Pots were watered daily using an automatic watering system and the duration was increased with seedling growth to maintain adequate soil moisture. Seven days after planting, seedlings were inoculated with approximately 1000 vermiform reniform nematodes of a local field collected Mississippi isolate MSRR04 [39], which had been maintained on tomato (*Solanum lycopersicon* cv. Rutgers). Approximately 28 days after inoculation, individual plants were removed from pots and the root system was gently agitated in tap water to remove soil. Roots were stained with red food coloring [40] and the number of swollen females attached to the root system were counted. To compensate for differences in root size, root samples were placed on paper towels to remove excess moisture and weighed with results expressed as the number of females per gram of fresh root weight. Because the accessions were screened as part of the breeding program and not evaluated in a single test, the mean level of infection for each accession was expressed as a female index (FI) value [41]; where the infection response is expressed as a percentage of the average number of females infecting the root system of the susceptible control included in each test. Accessions with FI values less than 10% were classified as resistant, moderately resistant with values ranging from 10 to 30%, moderately susceptible with values ranging from 31 to 60%, and susceptible with index values greater than 60%.

### Genotyping of *G. arboreum* accessions

One self-pollinated seed for each accession was planted in the greenhouse and leaf samples were collected from each accession 30 days after planting. DNA was extracted from the 246 accessions using the DNeasy Plant Mini Kit (Qiagen, Valencia, CA) following the manufacturer’s protocol. Quantification of the DNA samples was conducted using the Quant-iT PicoGreen dsDNA Assay Kit (Molecular Probes, Inc., Eugene, OR) with DNA quality assayed by electrophoresis of a 1 uL sample on a 1% agarose gel using 1× TBE running buffer.

GBS was performed at the Institute for Genomic Diversity, Cornell University, Ithaca, New York as described by Elshire et al. [42]. Restriction enzyme *Ape*KI was used for the preparation of DNA libraries. SNPs were identified using the TASSEL-GBS pipeline [43] in TASSEL: 3.0.166 following the method described in Li and Erpelding [38]. Briefly, raw fastQ sequences were trimmed of barcodes and reads from the fastQ files were collapsed into one master TagCounts file containing unique tags along with their associated read count information. These unique tags were then mapped to the *G. arboreum* (cv. Shixiyal, SXY1) reference genome [44] by Burrows-Wheeler Aligner [45] and tags that aligned to unique positions on the genome were retained for SNP calling. SNP discovery was performed for each set of tags that aligned to the exact same starting genomic position and strand. The genotype of the SNP was then determined by the default binomial likelihood ratio method of quantitative SNP calling in TASSEL: 3.0.166 [43]. Additional filtering steps were conducted in TASSEL to generate SNPs for GWAS using minimum minor allele frequency of 0.05, minimum taxa coverage of 0.8, minimum site coverage of 0.1, and maximum heterozygosity ratio of 0.2.

### Association mapping and linkage disequilibrium

GWAS was performed using the compressed mixed linear model (CMLM) [46] implemented in Genome Association and Prediction Integrated Tool (GAPIT) [47]. Both kinship, calculated using the Efficient Mixed-Model Association (EMMA) method, and principle component (PC) analyses were conducted in GAPIT to correct for false-positive associations from familial relatedness and population structure. The optimal number of PCs for GWAS was determined by activating the Bayesian information criterion (BIC)-based model selection in GAPIT. To correct SNP genotype significance level for multiple testing, a permutation test was performed. In brief, phenotypic values for the different accessions were swapped 1000 times. Association tests were ran on the original genotypic dataset with each phenotypic dataset harboring swapped trait values. Then, *P*-values for the most significant SNPs from each association test were extracted and ordered from smallest to largest. *P*-value thresholds for the genome-wide significant SNPs were decided as the 50th of the 1000 *P*-values (i.e. 0.05 threshold) generated from association tests. Genome-wide LD for linked loci (loci on the same chromosome) was calculated in TASSEL 5.0 [43] in terms of squared allele frequency correlation (r^2^). Decay of LD was displayed by calculating the averages of r^2^ values between the linked pairs of SNP loci with physical distance from 0 to 2000 kb using a custom Perl script. The Manhattan and quantile-quantile plots were generated using R package qqman [48]. The LD plot for significantly associated SNPs was constructed with Haploview software 4.2 [49].

### Allelic effects

Estimates of allelic effects generated by the linear model of GAPIT were examined for the significantly associated SNPs to determine the resistance allele. Resistance alleles are those having negative allelic effects on FI values. The additive effects of the identified significantly associated SNPs were examined by calculating the Pearson product-moment correlation coefficient between the number of resistance alleles carried by each accession and their respective reniform nematode resistance level (in terms of FI values). Figures and statistical tests were generated and performed in R.

### Candidate genes of reniform nematode resistance

The *G. arboreum* reference genomic sequences along with its gene model information were retrieved from the Cotton Genome Project database (http://cgp.genomics.org.cn/page/species/index.jsp). Candidate genes were obtained by extracting gene models within 72 kb of the significantly associated SNP, which is the total contig N50 length for the published reference genome [44]. Expression levels of the identified candidate genes in different *G. hirsutum* genotypes with and without reniform nematode infestation were retrieved from Li et al. [29].

## Results

### Phenotypic variation in reniform nematode resistance

**Fig. 1:**
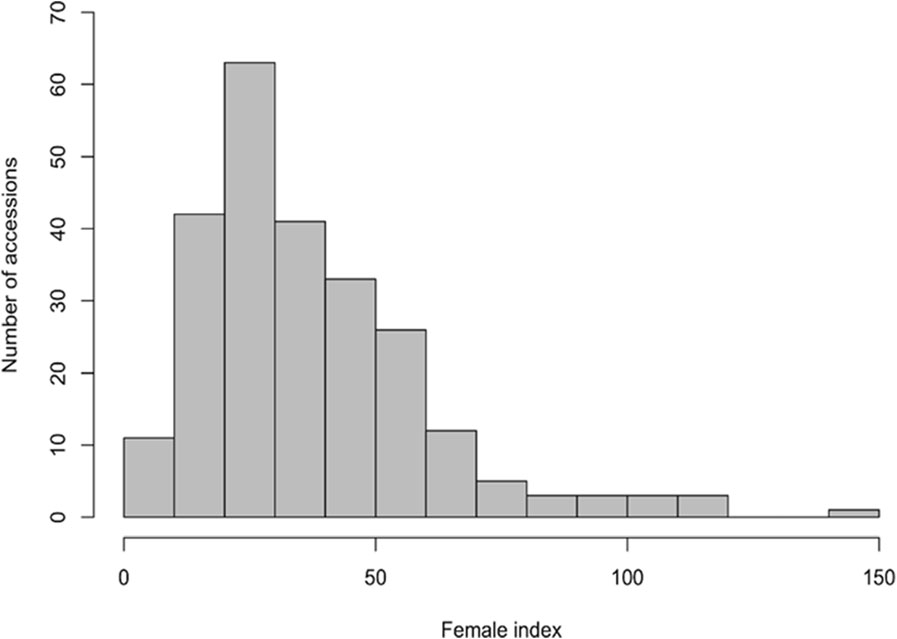
Frequency distribution of female index (FI) values for the 246 *Gossypium arboreum* germplasm accessions. FI = (average number of female reniform nematode on an accession per gram of root tissue / average number of female reniform nematode on the susceptible *Gossypium hirsutum* control genotype PI 529251 per gram of root tissue) * 100

RN resistance based on FI values relative to the RN susceptible *G. hirsutum* control genotype revealed a broad range in disease response with values from 2.8 to 140.3 for the 246 *G. arboreum* accessions (Additional file 1: Table S1). An approximate continuous distribution of RN resistance was observed with more accessions identified as moderately resistant (43%) or moderately susceptible (41%) compared to accessions categorized as resistant (4%) or susceptible (13%) (Fig. 1).

### Linkage disequilibrium, genome-wide association study, and allelic effects

**Fig. 2:**
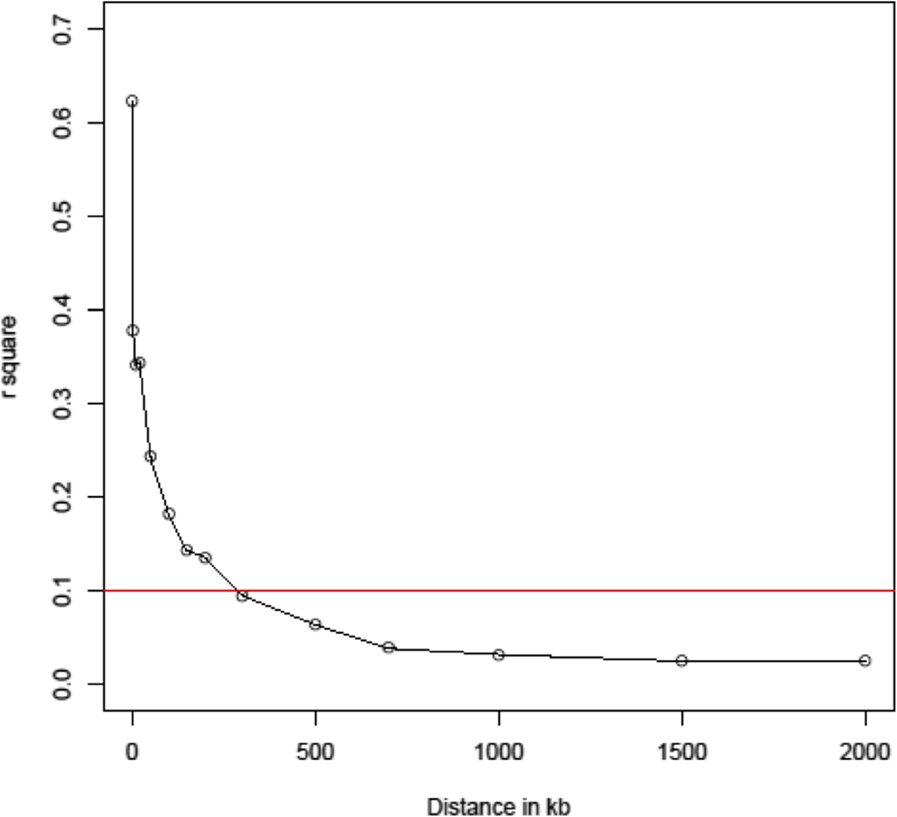
Genome-wide linkage disequilibrium (LD) pattern for the paired loci on the same chromosome. The red horizontal line indicates that LD (r^2^) decayed to 0.1 when the physical distance between a pair of loci reached 300 kb

Using 7220 SNPs (Additional file 2: Table S2), a mean pairwise r^2^ of 0.27 for the 13 chromosomes was determined. With an increase in the physical distance between the pair of SNPs, r^2^ decreased (Fig. 2). LD decayed to half of the maximum value of r^2^ at a distance of ~ 20 kb, to a value of 0.2 at a distance of ~ 100 kb, and to 0.1 when the distance between the pair of SNPs reached ~ 300 kb.

**Fig. 3:**
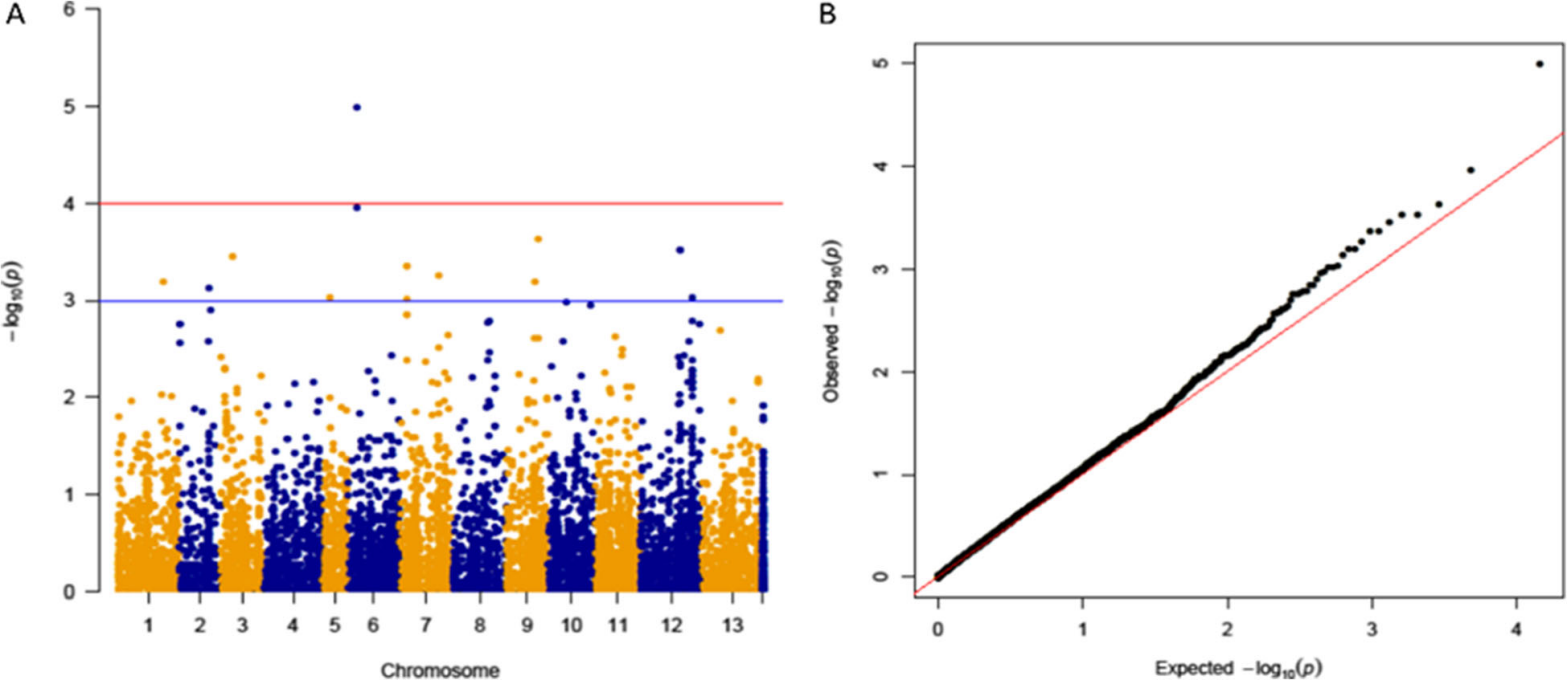
Marker-trait associations for reniform nematode resistance evaluated for the 246 *Gossypium arboreum* germplasm accessions. **a** Genome wide Manhattan plot of association from compressed mixed linear model; x-axis showed the SNPs along each chromosome; y-axis was the –log10 (*P*-value) for the association. S1_842793872 and S1_842793960 on chromosome 7, S1_634724647 and S1_634724595 on chromosome 12 are physically close to each other with nearly the same *P*-values (Additional file 4), so they overlapped as one dot in the figure. The alternate orange and blue dots represent SNPs mapped to different chromosomes, with the blue dots on the most right of the Manhattan plot located on scaffolds, which could not be assembled onto the 13 chromosomes of the *G. arboreum* reference genome. Dots above the blue horizontal line are SNPs with *P*-value < 0.001, whereas the one dot above the red line is the SNP with *P*-value < 0.0001. **b** Quantile-quantile (Q-Q) plot for the compressed mixed linear model

**Fig. 4:**
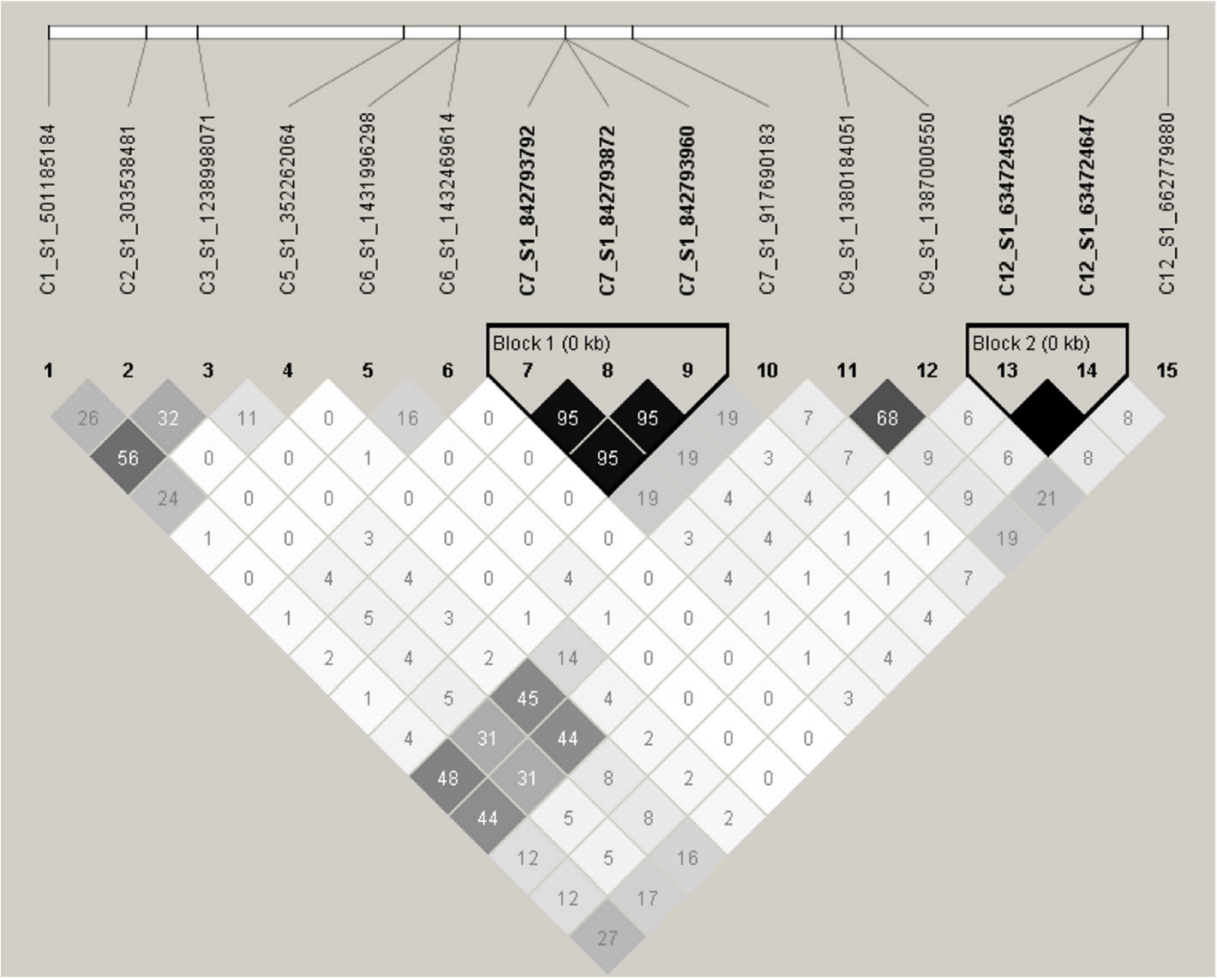
Linkage disequilibrium (LD) plot showing LD patterns among the 15 reniform nematode resistance-associated SNPs. The LD between the SNPs is shown in the intersection of the diagonals from each SNP. The numbers are based on D’ values and multiplied by 100. Values close to 0 indicate absence of LD (white shaded boxes), while those close to 100 indicate complete LD (black shaded boxes). The white block with black vertical lines on the top indicates the 15 SNPs on the reference genome (placed according to their physical positions on the reference genome). SNP markers on different chromosomes are listed, with their corresponding mapped chromosomes indicated as C1, C2, C3, C5, C6, C7, C9, and C12 in front of their ID. The two haplotype blocks (outlined in bold black line) indicate markers that are in high LD

GWAS was conducted using CMLM model with the optimal number of covariates included based on the results from model fitness test (Additional file 3: Table S3). Fifteen SNPs were identified as significantly associated markers with RN resistance based on a *P*-value threshold generated from permutation test (Additional file 4: Table S4; Table 1). The 15 SNPs were distributed over 8 chromosomes with one SNP on chromosomes 1, 2, 3, and 5, two SNPs on chromosomes 6 and 9, three SNPs on chromosome 12, and four SNPs on chromosome 7 (Table 1, Fig. 3a). One of the genomic regions (S1_1431996298) on Chromosome 6 showed the highest peak and explained 15% of the phenotypic variation, which was higher than the variation (~ 7.8%) estimated without this SNP. The phenotypic variation explained by the other SNPs ranged from 4.2 to 5.8% (Table 1). Local LD analysis for all significantly associated SNPs showed that markers on Chromosome 7 and 12 were in high intra-chromosomal LD, and that the 15 SNPs could be assigned to 12 loci (Fig. 4). The quantile-quantile plot (Fig. 3b) showed an upward deviation from the linear line around −log_10_ (*P*) = 3 indicating true positives for the 15 SNPs.

**Table 1:** Candidate gene data for the 15 SNPs significantly associated with reniform nematode resistance identified from genome-wide association study

Marker	Chr	Position	Candidate gene	Description	R^2a^	R^2^
S1_1431996298	6	21296008	Cotton_A_17782	DNA-directed primase polymerase protein isoform ×1	0.08	0.15
S1_1432469614	6	21769324	Cotton_A_17731	cct motif family protein	0.08	0.14
S1_1387000550	9	72903560	Cotton_A_02972	chaperonin cpn60- mitochondrial	0.08	0.13
S1_634724595	12	93797765	Cotton_A_06029	pentatricopeptide repeat-containing protein at1g16830	0.08	0.13
S1_634724647	12	93797817	Cotton_A_06029	pentatricopeptide repeat-containing protein at1g16830	0.08	0.13
S1_1238998071	3	27838614	Cotton_A_03279	coproporphyrinogen iii oxidase	0.08	0.13
S1_842793872	7	18184817	Cotton_A_14237	leucine-rich repeat protein kinase family	0.08	0.13
S1_842793960	7	18184905	Cotton_A_14237	leucine-rich repeat protein kinase family	0.08	0.13
S1_917690183	7	93081128	Cotton_A_29428	apo protein chloroplastic-like	0.08	0.12
S1_501185184	1	107382510	Cotton_A_24022	restin homolog	0.08	0.12
S1_1380184051	9	66087061	Cotton_A_12928	cytochrome p450 86a1	0.08	0.12
S1_303538481	2	70449510	Cotton_A_00283	wound-induced protein 1-like	0.08	0.12
S1_662779880	12	121853050	Cotton_A_18631	eukaryotic translation initiation factor 2 family protein isoform 2	0.08	0.12
S1_352262064	5	18150534	Cotton_A_17988	pre-mrna-processing factor 6-like	0.08	0.12
S1_842793792	7	18184737	Cotton_A_14237	leucine-rich repeat protein kinase family	0.08	0.12

*Chr* chromosome, *R*^*2*a^ R square of model without SNP, *R*^*2*^ R square of model with SNP

**Fig. 5:**
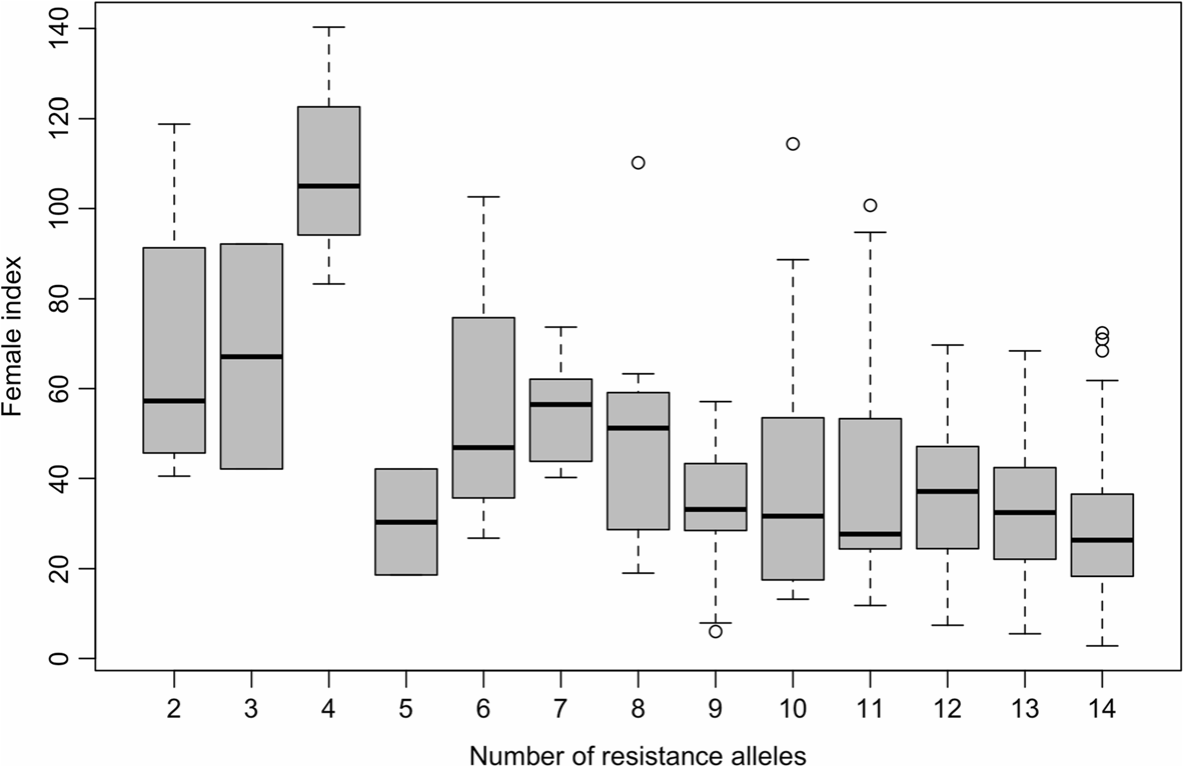
Female index (FI) for *Gossypium arboreum* accessions carrying the specific number of reniform nematode resistance alleles. The gray box represents the 25th through 75th percentile with the black bar representing the mean FI value for each of the gene models. The range in FI values is represented by the dashed line. Fewer than five accessions comprised each of the gene models with less than seven resistance alleles. Two accessions were represented in the gene models having three or five resistance alleles

The allelic effect estimates generated by the linear model of GAPIT showed major alleles as the resistance alleles except for S1_352262064. Additionally, additive effects were observed for RN resistance in *G. arboreum* with a negative correlation between the number of resistance alleles carried by each accession and their respective FI value (correlation value of − 0.43, *P*-value < 0.001), which resulted in higher RN resistance levels observed for accessions carrying a greater number of resistance alleles (Fig. 5). The majority of these accessions had six or more resistance alleles with only 11 accessions having less than six alleles. Two accessions were observed for each gene model having 3 or 5 resistance alleles.

### Candidate genes for reniform nematode resistance

Twelve candidate genes for RN resistance were identified by searching genes at or physically close to the detected significantly associated loci (Table 1). These 12 genes fell into various biological pathways, including redox reaction, signaling, translation, RNA processing and binding, and response to stress and wounding. By searching 72 kb regions flanking the significantly associated SNPs on the *G. arboreum* reference genome [44], 146 annotated genes were detected (Additional file 5: Table S5). By comparing the 146 genes to the differentially expressed genes (DEGs) [29], 18 out of the 146 genes were found to be significantly DEGs (fold change value > 2 and FDR *P*-value < 0.01) in response to RN infection (Additional file 6: Table S6).

## Discussion

LD has an essential role in GWAS. The distance between loci in which LD persists determines the number and density of markers needed for a reasonable resolution of association analysis [50]. Many factors affect the extent of LD, including genetic diversity, population size, admixture level, selection and mating pattern, as well as marker system [50, 51]. The LD decay estimates for predominantly self-pollinated species, such as cotton, are much higher compared to the extent of LD in out-crossing species such as maize (1–100 kb) [50]. As an example, LD decayed within 250 kb for *Arabidopsis thaliana* [52]; 75 to 500 kb for rice [53] and 100 to 600 kb for soybean [54]. A genome-wide LD extent level of 25 cM at a significance threshold of r^2^ = 0.1 was reported for *G. hirsutum* using SSR markers [34]. In the present study, LD decayed within ~ 300 kb at r^2^ = 0.1, which is similar to other self-pollinated species, but lower than *G. hirsutum* based on SSR markers. The variation between the cotton species investigated in the two studies and the marker systems employed would be factors contributing to the different rates of LD decay observed. In comparison, Su et al. [55] reported a mean LD decay distance of 100 kb with a mean marker density of 1 SNP per 24.85 kb for the 81,675 SNPs used in the study. Based on the extent of LD and marker density (1 SNP per 200 kb) in the present study, some loci may be left untagged, especially genomic regions with low local LD and few SNPs. The relatively low genome wide marker density in the present study can result from sequencing errors along with the high rate of missing data that frequently occur in GBS assays [43]. Genetic imputation can boost the number of SNPs by predicting genetic variants that were not observed for individual genotypes by using a reference panel or by estimating haplotype from observed genotype data. However, when a reference panel is not available such as the case with *G. arboreum*, a high false positive rate of imputation and minimal differences between imputed and non-imputed data could introduce an ascertainment bias to GWAS [56]. In the present study, non-imputed data were therefore used for GWAS.

The genome scan showed several peaks of moderate significance rather than major dominant peaks. This relative weak association may be the result of a narrow genetic base for the *G. arboreum* accessions used in this study [38] and the phenotypic variation typically observed in assaying for nematode resistance [9, 57]. While population structure and genetic relatedness can contribute to these results, it also suggested that RN resistance in *G. arboreum* is a complex trait conferred by multiple genes, where each gene contributes a moderate level of resistance. Although major genes for RN resistance have been reported [9, 11, 13, 22], extensive quantitative variation is frequently observed in segregating populations [9, 10]. This result also suggests that compared to maker-assisted selection, genomic selection might be a better strategy to take for breeding lines with higher RN resistance levels, which aims to utilize genome wide molecular markers and phenotypic data in large breeding populations to predict breeding values with high accuracy before they are field tested [58]. However, the difficulty in transferring RN resistance between cotton species and the inability to assay resistance for large populations could limit this approach.

The 15 SNPs that showed significant associations with RN resistance were distributed over 8 chromosomes. The occurrence of multiple genes and the greater frequency of resistant accessions in the *G. arboreum* germplasm collection would suggest this collection is a key source of RN resistance for cotton improvement. These significantly associated loci were not mapped to the same loci or homeologous loci as reported for the five RN resistance QTL introgressed into *G. hirsutum* [11, 13, 22] and these new sources of resistance would be of A-genome origin. Additionally, major alleles were identified for 14 loci that associated with the resistant phenotype indicating major alleles may have better molecular fitness in regard to RN resistance. The additivity of the resistance loci detected from this study, along with the fact that these loci represent novel genetic resources for RN resistance, suggest that higher RN resistance levels should be achieved by pyramiding resistance alleles identified from *G. arboreum* with alleles from other genetic resources. Genetic inheritance studies would support this conclusion as plants with higher levels of resistance than the parents have been observed in segregating populations [9, 10]. The occurrence of transgressive segregation in mapping populations has also been reported for root-knot nematode resistance [27].

Several biological pathways were suggested to be involved in RN resistance for *G. arboreum*, by assessing the gene annotation and gene ontology term for the 146 genes co-localized within the genomic regions of the 15 significantly associated SNPs. The closest gene model to the three SNPs on chromosome 7, which were in high intra-chromosomal LD, belongs to the leucine-rich repeat (LRR) protein kinase family. One class of genes in this family encoding LRR domain containing receptor like kinases (LRR-RLKs), constitute a main type of *R* genes involved in plant immunity responses [59]. Upon sensing invading pathogens, *R* genes can activate a series of downstream defense-related responses leading to plant resistance. In cotton, some *R* genes conferring resistance have been reported for plant-nematode interactions [60]. *R* genes were also suggested to mediate different levels of RN resistance through active regulation of expression levels [29]. The result presented here would further support the suggestion that *R* genes might be critical regulators in cotton resistance to RN, although further functional molecular studies are required to confirm this hypothesis. The SNP S1_1380184051 on chromosome 9 co-localized with a gene encoding cytochrome P450, which showed differential expression in response to RN infestation [29]. Cytochrome P450 enzymes function in the biosynthesis of many secondary metabolites such as gibberellins, abscisic acid, and other plant defense compounds [61]. Additionally, one gene encoding heat shock protein (HSP) 20 that co-localized with the two loci on chromosome 9 also showed differentially expression [29], suggesting its potential role in RN resistance. Other possible RN resistance candidate genes identified in this study included WRKY transcription factor coding genes co-localized on chromosomes 6 and 12, an ethylene responsive transcription factor (ERF) coding gene on chromosome 6, and *GRAS* transcription factor on chromosome 3. WRKYs regulate the expression of defense related genes in plant immunity responses [62] and in *G. hirsutum* their accumulation levels were dynamically regulated in response to RN infection [29]. Pathogenesis-related ERFs have been shown to be involved in soybean cyst nematode resistance responses [63, 64].

The gene expression analysis data used in this study were generated from *G. hirsutum*, which used the published genome of *G. arboreum* [44] as the reference A sub-genome for transcriptome assembly and read mapping [29]. By integrating this gene expression data, more insight on the possible genes and pathways associated with RN resistance in the A-genome background could be achieved, although the gene expression pattern in *G. arboreum* could be different compared to *G. hirsutum* as a result of genetic divergence during genome evolution. In the future, it is desired to include gene expression data from *G. arboreum* accessions with differential levels of RN resistance, so that genes exhibiting differential expression associated with RN resistance could be inferred as possible cis-regulatory elements affecting RN resistance. The inclusion of gene expression data could also help correct for false positives, because the quality of the reference *G. arboreum* genome is known to be relatively low, which could pose potential problems on read mapping. That is, the genomic physical positions of the identified SNPs could be inaccurate. Nonetheless, SNPs and potential candidate genes identified from this study will be a useful resource for further characterization of RN resistance in *G. arboreum* to aid in the transfer of resistance to *G. hirsutum*.

## Conclusions

High-throughput GBS data integrated with phenotypic data for 246 *G. arboreum* accessions with diverse RN resistance levels resulted in the identification of 15 SNPs representing 12 loci distributed over eight chromosomes that significantly associated with RN resistance. Major alleles were associated with resistance for 14 SNPs. This would indicate natural selection for RN resistance and/or selection during the breeding of *G. arboreum* germplasm, such as the selection of varieties more tolerant to environmental extremes. Also, additive effects were observed for RN resistance with accessions typically having more than five alleles; thus, higher resistance levels should be achievable by pyramiding resistance alleles from multiple sources. This would suggest that the accumulation of resistance alleles may be advantageous. Because breeding for RN resistance is hindered by the difficulty of introgressing resistance from *G*. *arboreum* and the lack of rapid procedures to assess resistance in large populations, the SNPs identified in this study or the identification of SSR markers in the associated regions would benefit marker-assisted selection approaches for the development of RN resistant varieties and the pyramiding of multiple resistance alleles. Populations have been developed for the RN resistant accessions to further characterize the genetics of resistance and for QTL mapping. As such, it would be obvious to someone working in the field of plant breeding to use the reported SNPs or to develop additional molecular markers in the associated regions identified in this study in order to breed elite upland cotton varieties with RN resistance.

Compared to the known loci involved in cotton RN resistance, the 12 loci identified in this study represent novel genetic resources. A survey of genes that co-localized with the RN resistance-associated loci identified 146 putative RN resistance candidate genes. These genes were involved in a wide range of biological pathways that may play a significant role in resistance. A cluster of LRR domain containing kinase genes, representing one type of *R* genes, were detected in the vicinity of three RN resistance-associated SNPs on chromosome 7. This result, together with the finding that RN resistance loci co-localized *R* genes had a significant higher accumulation level in resistant *G. hirsutum* genotypes, suggested the critical regulatory role of *R* genes in cotton resistance to RN. However, additional experiments need to be conducted to test the trait-loci associations identified for RN resistance in this study. RNA-seq experiments should be conducted for *G. arboreum* accessions to further explore the expression pattern of genes near the potential resistance loci and to identify candidate genes responsible for RN resistance.

## Additional files

Additional file 1: The 246 *Gossypium arboreum* germplasm accessions selected for genome-wide association study including the reniform nematode female index value for each accession. (XLSX 23 kb)

Additional file 2: The 7220 SNPs used for linkage disequilibrium analysis and genome-wide association study including major and minor alleles. SNPs highlighted in yellow identify chromosomal regions associated with reniform nematode resistance. (XLSX 6162 kb)

Additional file 3: Bayesian information criterion (BIC) values of compressed mixed linear model with different numbers of principle components used for association analysis in GAPIT. (XLSX 8 kb)

Additional file 4: Genome-wide association results for the 7220 SNPs included in the study. (XLSX 564 kb)

Additional file 5 The 146 annotated genes within 72 kb of the 15 significantly associated SNPs. (XLSX 19 kb)

Additional file 6: The 18 genes out of the 146 genes that were significantly differentially expressed in *Gossypium hirsutum* in response to reniform nematode infestation. (XLSX 13 kb)

## Abbreviations

BIC: Bayesian information criteria
CMLM: Compressed mixed linear model
DEG: Differentially expressed gene
EMMA: Efficient mixed-model association
ERF: Ethylene responsive factor
FI: Female index
GAPIT: Genome Association and Prediction Integrated Tool
GBS: Genotyping-by-sequencing
GWAS: Genome-wide association study
HSP: Heat shock protein
LD: Linkage disequilibrium
LRR: Leucine-rich repeat
MMT: Million metric tons
PC: Principle component
QTL: Quantitative trait loci
RN: Reniform nematode
SNP: Single nucleotide polymorphism
SSR: Simple sequence repeat
